# Editorial: Modality and language acquisition: how does the channel through which language is expressed affect how children and adults are able to learn?

**DOI:** 10.3389/fpsyg.2023.1334171

**Published:** 2023-12-04

**Authors:** Richard P. Meier, Christian Rathmann, Aaron Shield

**Affiliations:** ^1^Department of Linguistics, The University of Texas at Austin, Austin, TX, United States; ^2^Department of Deaf Studies & Sign Language Interpreting, Humboldt-Universität zu Berlin, Berlin, Germany; ^3^Department of Speech Pathology & Audiology, Miami University, Oxford, OH, United States

**Keywords:** modality, language acquisition, sign language, L2 acquisition, spoken language, multimodality, gesture, deafness

The most fundamental way in which human languages vary—their most essential typological dimension—lies in their “modality” of production and perception. Human languages may be spoken or signed, and perceived through hearing, vision, or touch. Oral-aural and visual-gestural languages are the native languages of substantial communities; tactile-gestural linguistic systems include the now-emerging languages of deaf-blind communities (Edwards, 2014; Edwards and Brentari, 2020; in this Research Topic, see Villwock and Grin, for a review of the perception of touch in sighted deaf individuals and deaf-blind individuals). That languages exist in these three modalities, or transmission channels, is testament to the plasticity of the human language capacity, and to its resilience.

In this Research Topic, our contributors examine a number of hypothesized differences between the visual-gestural and auditory-vocal modalities. “Modality differences” between languages are attributable to the differing resources and constraints of their respective transmission channels. For example, given the affordances of the visual-gestural modality, iconicity – the motivated, non-arbitrary relationship between a linguistic symbol's form and its meaning – appears to be more frequent in signed than in spoken languages; the role of iconicity in the learning of signed languages is examined here in Gappmayr et al., Hofweber et al., and Kurz et al.. Attention to iconicity in the sign literature may have been one factor that has pushed researchers on spoken languages to recognize that not everything is arbitrary in speech (e.g., Dingemanse et al., 2015).

Another property of signing has no obvious analog in speech. In sign, the manual articulators are the object of perception, unlike the oral articulators, which are largely hidden from view. One consequence is that many signs look quite different from the addressee's perspective than the signer's (Shield and Meier, 2018). Shield et al. argue that this phenomenon contributes to a distinctive characteristic (palm reversals) of the signing of deaf autistic children.

The phonological and morphological organization of signs appears to be more simultaneously-, and less sequentially-, structured than are the words of spoken languages. Consistent with this typological generalization, Gu et al. find that sequential complexity, but much less so simultaneous complexity, is a source of difficulty in children's imitation of pseudosigns. Yet, as Loos et al. observe in their contribution, sequentially-organized structures appear in signing in places where we might have anticipated simultaneity, whether in children's acquisition of signed languages as first languages, in the emergence of new signed languages, or in the grammar and adult usage of established signed languages.

Multimodality is not just a manifestation of the plasticity of the human language capacity, as important as that is. Instead, learners and users confront it every day. Hearing, sighted users of spoken languages integrate visual information from co-speech gesture with the auditorily-presented speech stream. Adult hearing learners of a signed language are not just learning a second language, they are learning a language in a new modality; several contributions discuss these so-called L2M2 learners (Hofweber et al.; Schönström and Holmström; Kurz et al.; Watkins et al.; Joyce et al.). Spoken languages are not only presented auditorily, but can also be represented visually through writing. Deaf individuals often learn a spoken language primarily through its writing system, as Caldwell-Harris and Hoffmeister and Hänel-Faulhaber et al. observe in this Research Topic. For deaf learners, their acquisition of a first, signed language may enable success in the visual learning of a spoken language (Mayberry et al., 2002).

The issues of multimodality, iconicity and phonological-morphological organization have been widely discussed in research on second-language acquisition by hearing learners of a first signed language. For example, sign frequency and iconicity facilitate sign recognition, whereas individual differences in cognitive abilities and language learning background seemingly play no role (Hofweber et al.). There are novel findings reported here: (a) disability does not appear to impact the phonological discrimination and perspective-taking abilities of adult L2M2 learners (Joyce et al.), (b) nonlinguistic visuospatial skills, including visuospatial working memory and mental rotation skills, are predictive of success in sign-language interpreting programs (Watkins et al.), and (c) compared to L2M1 learners, L2M2 learners tend to have greater difficulty acquiring those parts of the lexicon that are specific to signed languages, such as depicting signs (Schönström and Holmström). Kurz et al. closely examined the use of four types of depicting signs in short narratives produced by L2M2 learners; these types showed different learning trajectories.

Within the field of first-language (L1) acquisition studies, three modality-related issues are explored in some detail in this Research Topic: visual attention, age of acquisition, and the effects of such linguistic properties as the phonological structure of words vs. signs. Novack et al.'s findings indicate that infants allocate their visual attention differently to people and objects depending on the modality of the language that is being used. Later in development, hearing children (aged 2–8 years) who were natively exposed to sign pay more attention to the face during the production of ASL signs than do sign-naive children, but not so during the production of non-linguistic grooming or of mime gestures (Bosworth et al.). Singleton and Crume show that deaf children of Deaf parents already have finely-attuned visual attention abilities by the time they start preschool, while deaf children of hearing parents do not. Adding to these findings, Tomaszewski et al. find that deaf children growing up in deaf families outperform deaf children from non-deaf families on measures of phonological, morphological, and syntactic competence in Polish Sign Language. In addition to considering the impact of language experience on somatosensory processing, Villwock and Grin point out that sensory deprivation plays a role in the highly variable acquisition experiences of deaf and deafblind children. Finally, Gu et al. discuss modality-related similarities and differences in children's phonological development by comparing results from pseudo-sign and pseudo-word repetition tasks. More cross-modal experimental approaches are needed and will enhance our understanding of modality-specific and modality-independent properties of language acquisition.

Modality of language has broader impacts in society. In the realm of education, Singleton and Crume observe that the enhanced visual-attention abilities of deaf preschoolers from Deaf families lead teachers to direct fewer attention-directing cues and more positive participation cues to them than to deaf preschoolers from hearing families, showing that early exposure to a signed language leads to better classroom interactions even in preschool. Despite the importance of the classroom as a site for sign learning, Goppelt-Kunkel et al. find that the presence of a single deaf peer or deaf educator in an inclusive kindergarten group is not sufficient to encourage hearing children in that classroom to use signs. Finally, Horton and Singleton examine the complex ways that modality of language affects the turn-taking skills of deaf children acquiring sign languages in a variety of settings, including the classroom.

Modality also has implications for the concept of neurodiversity, which in recent years has lifted discussions of atypical conditions from the realm of disorder and helped shift researchers to an appreciation of differences. Shield et al. consider how studying deaf autistic signers can inform our understanding of modality effects in signed and spoken languages, while Villwock and Grin point to the need for more research on the language acquisition of deafblind individuals in order to better understand the differential impacts of sensory deprivation vs. language experience on neuroplasticity and somatosensory processing. Lastly, Joyce et al. use the construct of disability to analyze the intersection of language, modality, and cognition, finding that the signed modality does not disadvantage neurodiverse learners.

Finally, we note that most of the authors who are published in this Research Topic have spent their careers working largely on signed languages. We had hoped to receive more submissions from researchers who work primarily on spoken languages. But we think too few researchers on spoken languages are delving into how the resources and constraints of the oral-aural modality may shape the organization of spoken languages. Researchers on spoken languages should, in our view, be more attentive to this problem. In contrast, the possible effects and non-effects of language modality are front and center in the sign literature, perhaps because all researchers working on signed languages are also familiar with spoken languages, or perhaps because spoken languages remain a default against which signed languages are inevitably compared. Indeed knowledge of the linguistics of spoken languages may sometimes skew our analyses of signed languages, thereby obscuring differences between sign and speech. In the future, we hope to see more attention to the effects of language modality on the structure and acquisition of language, not just by researchers on signed languages, but by researchers from across the language sciences.

## Author contributions

RM: Writing—original draft, Writing—review & editing. CR: Writing—original draft, Writing—review & editing. AS: Writing— original draft, Writing—review & editing.
